# “Can you keep it real?” : Practical, and culturally tailored lifestyle recommendations by Mexican American women diagnosed with type 2 diabetes: A qualitative study

**DOI:** 10.1186/s12912-017-0232-4

**Published:** 2017-07-06

**Authors:** Sandra Benavides-Vaello, Sharon A. Brown, Roxanne Vandermause

**Affiliations:** 1 Montana State University, College of Nursing, Missoula Campus, 32 Campus Drive #7416, Missoula, MT 59812-7416 USA; 2 The University of Texas at Austin, School of Nursing, Austin, TX 78701 USA; 3 University of Missouri-St. Louis, Seton 315, College of Nursing, South Campus, St. Louis, MO 6321-4400 USA

**Keywords:** Diabetes, Hispanic, Culture, Self-management, Contextual reality

## Abstract

**Background:**

The purpose of this article is to engage clinicians in a dialogue about ideas on how to provide more specific, contextually relevant, practical and culturally tailored diabetes self-management recommendations as suggested by Mexican-American women diagnosed with type 2 diabetes. Current diabetes self-management recommendations, targeting Mexican Americans in particular, remain largely broad (“reduce your calorie intake” or “cut back on carbs”), overly ambitious (“stop eating tortillas”), and relatively ineffective (Svedbo Engström et al., BMJ Open 6(3):e010249, 2016; Johansson et al., Int J Qual Stud Health Well-being 11, 2016; Oomen et al., The Diabetes Educ 25:220-225, 1999; Franek, Ont Health Technol Assess Ser 13(9):1-60, 2013; Purnell et al. Patient 9:349, 2016).

**Methods:**

A secondary and focused analysis (*N* = 12) was performed on data gathered from a larger qualitative study (*N* = 16), which explored diabetes among Mexican-American women residing in rural South Texas.

**Results:**

Findings from the secondary analysis were that study informants elicited more realistic or contextually relevant, specific self-management strategies that reflected the cognitive, emotive, and behavioral areas but were reframed within the context of the Mexican-American culture. Self-management strategies fell into the categories of: (a) environmental controls, (b) avoiding overeating, (c) lifestyle changes, (d) cooking tips, and (e) active self-management.

**Conclusions:**

Diabetes remains a serious health threat to Mexican Americans, women in particular. Few individuals attain glycemic control, likely due in part to the disconnect between global and non-contextual self-management recommendations offered by health care providers and the need for more detailed and realistic guidance required for the day-to-day self-management of diabetes.

## Background

Nearly 13% of Hispanic adults have type 2 diabetes (T2DM) and this population is 1.5 times more likely to die from the disease than non Hispanic Whites [1, 2]. However, among Mexican Americans, the largest Hispanic subpopulation, prevalence rates are even higher and are approaching 14% amongst adults 20 years of age and older [3]. For Mexican-American women, the threat of diabetes dominates their daily lives as they have higher prevalence rates than their male counterparts, and are more likely to experience risk factors such as obesity, being overweight, and having sedentary habits, compared to other women in general [4].

These factors and the complexity of diabetes and diabetes management present difficulties for both clinicians and patients. One of the most critical elements of diabetes self-management, but most challenging behaviors to tackle, is diet. The ways of food (foodways) are deeply entrenched in most cultures, including the Mexican-American culture. Therefore, diet is one of the major diabetes self-management components that is influenced by social and cultural elements, which synergistically play a large role in the ultimate health outcomes of affected persons [5]. Over the past 2 or 3 decades, we’ve made significant progress in diabetes self-management from the perspective of establishing the value to patient outcomes, particularly on glycemic control; identifying characteristics of successful, evidence-based programs; and obtaining Medicare benefits for DSME and medical nutrition therapy. However, diabetes disparities continue to plague minority groups who experience a greater diabetes burden. Clearly, we still have more work to do establish successful culturally tailored interventions for minority groups at high risk for the disease.

An estimated 80 to 95% of chronic disease management, including diabetes, occurs in an individual’s home or social environment [6, 7, 8]. Over time, persons with diabetes, or any chronic illness, are capable of developing considerable and contextually situated expertise in the management of their illness [6, 9–12]. For those with diabetes, proficiency can be reached within 5 to 15 years of being diagnosed with the disease [11, 13, 14]. Attaining expertise in diabetes self-management is a developmental and often non-linear process involving periods during which individuals with diabetes create and refine approaches for managing their illness, and these approaches are influenced by the realities of their environments. Approaches include: interpreting body cues, foreseeing threats to control, creating cooperative relationships with health care practitioners, and garnering support from significant others [9, 11, 15, 16]. Thus, self-management strategies are developed, shaped and influenced within sociocultural environments, as well as by knowledge of how one’s body responds to behavioral changes.

T2DM, as with other chronic illnesses, is a personal experience within a broader sociocultural network. Accordingly, symptoms and effective self-management strategies may depart from textbook standards [11, 13, 17]. However, the idea of the individual with diabetes as the expert in the day-to-day management of their care is often undervalued by some clinicians providing their care, due in part to the limited time that health care providers have during patient visits to teach the recommended self-management skills. The concept of “patient as expert” also conflicts with the reality that patients and health care practitioners represent different “cultures” and language. We have found that some health care providers have little confidence that patients will follow behavioral recommendations, particularly Latinas who have many cultural, family, and role challenges that may serve as barriers to diabetes self-management. Further, in some provider/patient situations there may be a predominant belief held by both the patient and the provider that management expertise lies in the health care professional [18]. Accordingly, lay or patient language, practical knowledge, and realistic strategies for diabetes self-management that are cultivated and employed by patients may be met with resistance, especially by clinicians firmly entrenched in biomedical model approach for managing diabetes.[19–23] Clearly, current efforts do not seem as effective as expected; research has demonstrated that despite the development of new technologies and self-management tools, less than 30% of persons with diabetes achieve recommended glycemic targets [24]. This low level of glycemic control is likely worse in minority populations who experience more barriers and challenges to disease self-management.

### Aim

The purpose of this article is to engage clinicians in a dialogue on how to identify more specific, practical, contextually situated, and culturally tailored diabetes self-management recommendations as suggested by low-income Mexican-American women diagnosed with T2DM. The vehicle employed for this dialogue is a focused analysis of self-management strategies developed by low-income Mexican-American women with diabetes who resided in rural South Texas. Presented here, and using participant language or *emic* dialect, are the findings of this focused analysis. Ultimately, the aim is to provide clinicians with patients’ perspectives of the type of practical and culturally oriented guidance needed, particularly around dietary management of diabetes.

## Methods

The secondary and focused analysis reported here is part of a larger qualitative and ethnographically oriented inquiry that examined the interrelationships among foodways (how food is selected, prepared, served and all related rituals), culture, and diabetes in rurally located Mexican-American women [25]. The primary study was conducted in rural South Texas, on the Texas-Mexico border, in one of the most impoverished communities in the U.S. Purposeful sampling methods were used to recruit 16 Mexican-American women diagnosed with T2DM. Observation and personal interviews with informants (participants) were the primary methods for collecting data. Six themes were generated from the data, including: La Dieta, Location and Fluidity of Food, Confidence-Defiance Self-Management Connection, Negotiating Sociocultural and Biomedical Expectations, Eating for Diabetes is a Family Affair, and *Strategies for Self-Management*. These themes are published and explicated elsewhere [25, 26].

*Strategies for Self-Management* is a salient theme chosen for extended exploration in this focused analysis because it illustrates current trends in chronic disease management. Results of the primary analysis of this theme pointed to specific approaches the experienced informants had developed in relation to managing changes in food habits while meeting social and cultural expectations. Twelve of the 16 informants had been diagnosed with diabetes for at least 10 years, and were considered experts or experienced in diabetes self-care because of their expressed commitment to wellness over time. Longevity of disease is linked with patient expertise or self-knowledge of disease trajectory, as patients are better able to deal with new or unexpected events [27–30]. Therefore, informant experience and insight were key to illuminating how low-income Mexican-American women in this part of the country successfully engage in self-management activities and provided a context within which these activities occur. Since the focus of the analysis was understanding person-centered experiences living with diabetes, the accounts of these women and not their biometrics informed our results.

A comprehensive menu of guiding questions was developed for the original study to elicit dialogue from the study informants [26]. One question, *“What sort of advice would you give other women who have diabetes and are having trouble making healthy food choices to care for their diabetes?*”, elicited substantial dialogue and generated much of the data used to develop the theme *Strategies for Self-Management* [25]. The strategies put forth by the informants, in their own words (*emic*), were developed over time, reflected a balanced approach, had been tested by informants, and provided them with a skillset that could be used to navigate through multifaceted sociocultural circumstances and yet carry out expectations of Mexican American women in this community. The sample size for this portion of the analysis is appropriate for eliciting common human experience in a population subset [31]. A detailed examination and analysis of the *Strategies for Self-Management* theme is presented here.

## Results

The informants created and employed strategies related to foodways, social ways, and home life, as well as within a broader socio-cultural context. The strategies pulled from cognitive, emotive, and behavioral arenas, but did so within the Mexican-American culture in rural South Texas and were stated in practical, specific, and concrete language. The more commonly articulated strategies by the women in this study are listed in Table 1 and have been organized into the categories of: a) environmental controls, b) avoiding overeating or stress eating, c) lifestyle changes, d) family, e) cooking tips and f) active self-management. The strategies shared by participants were not bound to a single category but were fluid and could be applicable to more than one category. Thus “drink more water” as a strategy could be placed under lifestyle change as well as avoiding overeating or stress eating.

**Table 1:** Strategies developed in managing their diabetes

Category	Strategies
Environmental Controls	o Make your own burgers and tacos o Cook more at home o Go to the grocery store alone o Avoid aisles in the grocery store that have the food items on sale that are bad for you o Remove temptation from your home o Not making tortillas helps because when you buy, once the package is gone, they are gone
Avoid Overeating/Stress Eating	o Don’t let yourself get hungry o Limit in between snacking o Keep active, walk o When you feel stressed or want to eat go for a walk o Drink more water o Satisfy the craving from the beginning so that you don’t keep eating foods you don’t really want
Lifestyle Changes	o (Eat) Foods better for you the sugar in their bodies o You can eat anything you want-even menudo-just limit the amount o Get into a routine o The progress takes time, don’t do everything at once, just step by step o Make separate meals o Smaller portions of the foods you love o Eat whole wheat flour tortillas o Get away from processed foods o Tortillas-flour, limit to once a week o Eat earlier in the evening
Family	o Put them (kids) first o (Think) Your family is going to be healthier o Go to the grocery store alone o They may not like it at first, just keep trying, think of what will happen to you o Set the table and serve the food, no choices o Put 2% or 1% milk in the whole milk container, keep the same label, family thinks they are drinking whole milk
Cooking Tips	o Don’t drink regular coke-try lemonade with Splenda® or water o Learn how to use the steamer, broiler, and oven o Stick with natural foods
Active Self-Management	o Ask (health care providers and other staff) what exactly you should eat to take care of your diabetes o See her doctor to make sure she is on the right medicines o See your dietitian o Take medicines religiously

### Environmental controls

Environmental controls were associated with managing food challenges in the home and social environment, such as the grocery store. These approaches included making burgers and tacos at home versus buying them from a restaurant (fast food or local eatery), going to the grocery store alone and avoiding aisles with unhealthy food sale items, and removing temptations from the home. One informant describes how she incorporated environmental controls necessary to making healthier food choices:

…And I think in my mind when I go sometimes to HEB® [grocery store chain] and I see candies like that different kind, and I just go around to another way. I stay off of there [that aisle] even for my kids. Sometimes they say, they beg, “Mom, get them because they’re on special.” And I say if I get them I did the wrong thing [for] them…

### Avoid Overeating/Stress Eating

Guidance by clinicians in relation to avoiding overeating, or avoiding eating as a way of managing stress are fundamental components of a dietary management plan. Nonetheless, these lessons or guidance are often provided using clinical nomenclature, stated in global terms, and lack contextual examples. In contrast, informants in this study proffered practical, specific solutions and strategies such as don’t let yourself get hungry; when you feel stressed go for a walk; and satisfying cravings from the beginning so that you don’t keep eating foods you don’t really want. One suggested approach to avoid overeating involved controlled submission to the desired food (“a craving”) as a strategy to avoid overeating, even if the food item was not considered a healthy choice.

I will usually have a little piece of candy. I’ll buy the bite size candies and I’ll just grab a Baby Ruth, which has the peanuts, I love the peanuts and the caramel. That’s about the only candy I usually buy, the Baby Ruth. …if you crave chocolate just eat [a] piece of it and the craving will go away. That’ll take my craving away and I’ll be fine after that …

### Lifestyle Changes

A common thread that ran throughout all categories was the notion of balance (*equilibrar)*, which is a key value in the Mexican-American culture and a major part of their health and social belief systems. This tacit norm was most present in the *Lifestyle Changes* category and recommended strategies included “eat anything you want-even *menudo*-just limit the amount,” progress takes time [so] don’t do everything at once, and limiting foods you love [e.g. tortillas] to once a week. Thus individuals would avoid feeling deprived while adhering to portion control, both of which were critical elements in this balanced approach.

Eat just a little bit of everything. Like when you have [prepare] a whole turkey, you don’t eat the whole turkey, you eat just a little bit not the whole turkey or the whole chicken, …[just] have a little bit. You’re going to go to Whataburger® or whatever, don’t have a big one [burger], have a small one….

### Family

Family and children are pivotal components in most Hispanic cultures, including Mexican Americans. In general, familial support and the inclusion of family in diabetes education and self-management programs result in better health outcomes [32, 33]. It was not surprising then that strategies identified by informants also revolved around family and included tactics such as “put them (kids) first,” “(think) your family is going to be healthier,” and “they may not like it at first, just keep trying, think of what will happen to you.” One of the informant’s stories embodies the value and importance of considering family when taking self-management actions:

And what I see in my family [is that] they're working at this stuff [eating healthier] too. They see that we're [family members with diabetes] going through this because I try to tell them. Let's start it [taking care of yourself] now because you are on another generation. By the time you are on your 40s maybe you're not going to make it to that age [due to diabetes complications]. …you see the problems [challenges managing diabetes] we're going through, and we don't want that to happen to you all. So the best thing is for you all to take care right now and not later.

### Cooking tips

Some of the most practical and specific guidance provided by the informants was related to the preparation of food. “Don't drink regular coke-try lemonade with Splenda® or water”; “learn how to use the steamer, broiler, and oven; and stick with natural foods [not processed]” are examples of the wisdom they wished to share. The women in the study recognized that carrying out matriarchal duties and responsibilities was an important part of their lives. However, several also felt that additional skills needed to be learned in order to be successful at managing their diabetes.

We’ve been trained and taught the skill of domesticism [how to be a domesticate]. It’s not cooking. They don’t know how to use the oven. I can literally say that I walk into hundreds and hundreds of homes here and start counting that the oven has never been used. They don’t know what it is to broil, they’ve never used a broiler, they don’t know what the broiler pan is for. They don’t know what it is to steam.

Thus it wasn’t enough to tell patients to steam or broil their food instead of frying; actual demonstrations that specifically addressed what is a steamer basket/broiler, what cultural foods can be steamed/broiled, and how to use steam/the broiler for cooking were considered the type of guidance needed for patients to be successful at diabetes self-management.

### Active self-management

The strategies included in the active self-management category were the most aligned with traditional clinical care nomenclature and used the broadest terminology. These included asking health care providers and other staff members what exactly you should eat to take care of your diabetes; seeing your doctor to make sure you are on the right medicines; seeing your dietitian; and taking medicines religiously. Even so the informants still used specificity (e.g. *what exactly you should eat to take care of your diabetes*) in their language.

…a lot of people take that [being on medication] for granted. “Oh, it’s okay [my diabetes], I’m on medication.” But you have to work for yourself. It’s not just the medication. When I go to the doctor I always ask questions, I’m never, you know [shy about asking questions]…

## Discussion

The work presented here infers that additional research, both qualitative and quantitative, is merited in this area. Qualitative studies that further explore the everyday and practical tactics employed by Latinas to manage their disease (strategies for self-management) can guide the development of needed quantitative inquiries. The latter would be larger in scale, possibly allow for generalization to other settings, and have the potential to yield larger data sets about the social and cultural factors influencing the control of diabetes in this specific study population. Further, qualitative studies are intended to provide rich contextual description and generate a greater understanding of the provincial milieu, versus a broader application of findings. Consequently, the focused exploration may be viewed as a limitation in that qualitative works cannot be generalized to other settings.

Nonetheless, this narrative can serve to better inform providers that adaptation of educational approaches to diabetes self-care needs to address the cultural context in practical and concrete ways to be most effective, and representatives from that culture can guide the design of materials and approaches to enhancing self-care support. Some statement might be useful as to how this evidence will be applied to diabetes self-care support for this population. A statement might also be warranted as to how much one can generalize to other populations from this sample of 12 Mexican-American women with diabetes who reside in an impoverished region of South Texas. The literature underscores the commonly discrete perspectives of providers compared to persons diagnosed with diabetes. Even so, practitioners and their patients who have the disease often approach diabetes from parallel orientations, the former from a biomedical model and the latter from a socially constructed model of illness. These positions are not necessarily diametrically opposed, but can create tensions between provider and patient, making successful management of diabetes a challenge. While the concept of provider-patient relationship is gaining acceptance in the management of chronic disease, clinicians and patients have different ideas of what such a relationship means. Clinicians’ expectations are that the patient will comply with the management plan, while the patient wants to receive guidance that is practical, specific, and that makes sense given their contextual realities. These different perspectives on daily management of diabetes can engender frustrating experiences between clinician and patient on particular aspects of care, such as dietary management.

## Conclusions

﻿The growing economic and social burden of diabetes among Hispanics calls attention to the need for alternative approaches in how self-management guidance and/or recommendations are provided. The intention of the work presented here was to begin a dialogue or discussion with clinicians about providing more practical, realistic, and culturally tailored diabetes self-management recommendations for persons with diabetes. The importance in the data presented here is the *emic* (insider/informant) diabetes-related language or dialect used and the perspectives shared of low-income Mexican-American women to manage T2DM in rural South Texas. It is the plain speak, the non-academic and non-biomedical nomenclature employed here that the *etic* (outsiders) should take note of and deliberate on how biomedical models of health delivery can incorporate more pragmatic, plain language, contextually situated and culturally tailored diabetes self-management guidance to persons with diabetes.

## Abbreviations

DSME: Diabetes self-management education
T2DM: Type 2 diabetes mellitus
U.S.: United States
